# Association of the COVID-19 pandemic with changes in objectively measured sedentary behaviour and adiposity

**DOI:** 10.1038/s41366-023-01274-9

**Published:** 2023-02-16

**Authors:** Keita Kinoshita, Naoki Ozato, Tohru Yamaguchi, Hiroto Bushita, Motoki Sudo, Yukari Yamashiro, Kenta Mori, Yoshihisa Katsuragi, Hiroyuki Sasai, Koichi Murashita, Yoshiko Takahashi, Kazushige Ihara

**Affiliations:** 1 Department of Active Life Promotion Sciences, Graduate School of Medicine, Hirosaki University, Aomori, Japan; 2 Health & Wellness Products Research Laboratories, Kao Corporation, Tokyo, Japan; 3 Department of Social Medicine, Graduate School of Medicine, Hirosaki University, Aomori, Japan; 4 Personal Health Care Products Research Laboratories, Kao Corporation, Tokyo, Japan; 5 Research Team for Promoting Independence and Mental Health, Tokyo Metropolitan Institute for Geriatrics and Gerontology, Tokyo, Japan; 6 Research Institute of Health Innovation, Graduate School of Medicine, Hirosaki University, Aomori, Japan; 7 Center of Innovation Research Initiatives Organization, Hirosaki University, Aomori, Japan

**Keywords:** Epidemiology, Obesity

## Abstract

**Background:**

Several studies have reported that the coronavirus disease (COVID-19) pandemic has increased sedentary behaviour and obesity; however, these analyses used self-reported data, and the association between sedentary behaviour and visceral fat and adipocytokines during the COVID-19 pandemic remains unclear. We aimed to investigate the association of the COVID-19 pandemic with objectively measured sedentary behaviour and these obesity-related factors.

**Methods:**

Longitudinal analysis was conducted on 257 Japanese participants who underwent health check-ups in 2018 before and in 2020 during the COVID-19 pandemic. For both time points, sedentary behaviour was measured using an accelerometer for at least 7 days, visceral fat area (VFA) was measured using abdominal bioelectrical impedance analysis, and blood adiponectin level was measured using latex agglutination turbidimetric immunoassay. Multiple linear regression was performed to determine the association between sedentary behaviour and these outcomes.

**Results:**

Compared with data in 2018, sedentary behaviour and VFA were significantly increased (*P* < 0.001, *P* = 0.006) whereas adiponectin level was significantly decreased (*P* < 0.001) in 2020. Increased sedentary behaviour was significantly associated with an increase in VFA (β = 3.85, 95% CI 1.22–6.49, *P* = 0.004) and a decrease in adiponectin level (β = −0.04, 95% CI −0.06 to −0.01, *P* = 0.005). However, the association of sedentary behaviour with adiponectin level was not significant after considering the effects of VFA.

**Conclusions:**

The COVID-19 pandemic was associated with objectively measured sedentary behaviour and obesity-related factors in Japanese adults. Additionally, an increase in sedentary behaviour was associated with an increase in VFA, whereas the association of sedentary behaviour with adiponectin was partly mediated by VFA. These results suggest that avoiding increasing sedentary time is important to prevent visceral adiposity thereby ameliorating adiponectin, especially during behavioural limitations such as the COVID-19 pandemic.

## Introduction

The incidence of obesity has increased over the recent decades [1]. The coronavirus disease (COVID-19) pandemic, caused by the SARS-CoV-2 virus, has worsened the obesity epidemic [2, 3] because of an inactive lifestyle. Obesity is recognised as the cause of severe health problems, such as cardiovascular disease [4] and increased COVID-19 severity [5]. Body mass index (BMI) is frequently used in clinical settings to assess general obesity status. Compared to BMI, visceral fat accumulation assessed as visceral fat area (VFA) is reported to be more strongly associated with cardiovascular disease [6, 7] and COVID-19 severity [8], which is partially because BMI cannot differentiate among visceral fat, subcutaneous fat, and muscle mass. One possible explanation for this difference between VFA and BMI is that adipocytokines such as adiponectin, which are reported to have a protective role in both cardiovascular disease [9] and COVID-19 severity [10], are more closely related to visceral fat than to BMI [11]. Several studies have reported that the COVID-19 pandemic has caused weight gain; [12, 13] however, no study has investigated how the COVID-19 pandemic influences visceral fat accumulation and adipocytokines.

Obesity is caused by an imbalance between energy intake and expenditure, and sedentarism causes obesity by decreasing energy expenditure [14]. Previous studies reported a significant association between sedentary behaviour and obesity-related factors like VFA and adiponectin levels before the COVID-19 pandemic [15–20]. Since the WHO declared the COVID-19 pandemic in March 2020, behavioural limitation measures, such as lockdowns, have been implemented in various countries worldwide, and health behaviours have changed [21, 22]. In Japan, although a State of Emergency was declared on 7 April 2020 and regional restrictions were implemented, the SARS-CoV-2 infection rates in 2020 were low and mandatory lockdowns were not imposed [23]. For example, people in Aomori prefecture, a rural area of Japan, were instructed to refrain from nonessential outings/travel crossing prefectural borders, while relying on their self-restraint. Several studies have reported increased sedentary time and decreased physical activity, such as daily steps, due to the COVID-19 pandemic [12, 13, 24] and reported a significant association between sedentary time and physical activity with obesity [25, 26]. However, these studies have a potential bias because sedentary behaviour was assessed subjectively instead of using objective methods such as accelerometery, which is currently regarded as the gold standard [27]. There is a lack of research on how objective measures of obesity-related factors changed before vs. during the COVID-19 pandemic.

Due to the nature of behavioural measures, sedentary behaviour may have been much more affected by the COVID-19 pandemic compared to other health behaviours [13], thereby decrease in energy expenditure through several pathways, such as reduced physical activity and muscle mass. Therefore, we investigated the association of the COVID-19 pandemic with the changes in objectively measured sedentary behaviour and adiposity. We also investigated the association between objectively measured sedentary behaviour and adiposity using population-based health check-up data collected before and during the COVID-19 pandemic.

## Methods

### Participants

The Iwaki Health Promotion Project was launched in 2005. As part of the project, an annual health check-up was conducted for adults living in the Iwaki region of Hirosaki City, Aomori Prefecture, located in northern Japan [28]. Aomori Prefecture has the shortest life expectancy among all the 47 Japanese prefectures, mainly due to unhealthy lifestyles (e.g., a high rate of smoking, a high percentage of obesity, and heavy drinking). In 2015, the life expectancy of people in Aomori Prefecture was 78.7 years for men and 85.9 for women, which was the lowest in Japan—the national average 80.8 and 87.0 years, respectively—but still higher than the global average (71.8 years). All adult residents (approximately 6000 people aged ≥20 years) in this region were invited based on their resident registrations. We included all adults (aged ≥20 years) who attended the health check-up and excluded those who did not provide written informed consent in this study. The present longitudinal analysis was performed using data obtained from the 2018 health check-up before the COVID-19 pandemic and the 2020 health check-up conducted during the COVID-19 pandemic.

In 2018, 1,056 individuals participated in a health check-up from May 27 to June 5. In 2020, 524 individuals participated in a health check-up from September 17 to September 25. The number of participants in the 2020 check-up was approximately half that in a typical annual check-up because of the COVID-19 pandemic. Of these, 410 individuals who participated in both the 2018 and 2020 health check-ups were enroled in the study. We excluded 153 participants due to incomplete clinical assessments, dietary data, or accelerometer data. Ultimately, 257 participants were included in the analysis. Supplementary Figure 1 shows the number of participants at each time point. Participants who were excluded from the analyses were more likely to be younger, have higher alcohol intake, or/and were current smokers compared to those included in the analyses (Supplementary Table 1).

The study was approved by the Ethics Committee of Hirosaki University School of Medicine (2018-012, 2018-063, and 2020-046-1) and was conducted in accordance with the principles of the Declaration of Helsinki. Written informed consent was obtained from all participants before the study. This study was registered in the University Hospital Medical Information Network (https://www.umin.ac.jp) prior to the analyses (UMIN ID: UMIN000036741).

### Sedentary time and physical activity

Sedentary time and physical activity were measured using an accelerometer, HW-100 (Kao Corporation, Tokyo, Japan), in 2018 and HW-200R (Kao Corporation, Tokyo, Japan) in 2020. The HW-100 and HW-200R are similar, with the HW-200R being more compact than the HW-100. The epoch length of the accelerometer was 4 s with a sampling frequency of 64 Hz. Activity intensity was measured as previously described [15, 29, 30]. Briefly, accelerometer data were calculated as the time spent in each of the following intensity levels: 1) sedentary behaviour, ≤1.5 metabolic equivalent tasks (METs); 2) light physical activity, 1.6–2.9 METs; and 3) moderate-vigorous physical activity, ≥3 METs. A period of ≥35 min, during which activity was not recorded using an accelerometer, was designated as non-wear time. The sedentary time was expressed as the mean daily hours across all adherent days (wearing ≥10 h/day) for all participants. For physical activity, the number of steps taken was also measured, expressed as the mean number of daily steps on all adherent days.

The participants were instructed to wear the accelerometer on the waist throughout their awake period, except during swimming or bathing, and to maintain their usual activities. Additionally, the participants were instructed to begin wearing the accelerometer promptly after completing their health check-up and to return it after 10 days. The criterion for analysis was wearing the accelerometer for a total duration of ≥7 days (≥10 h/day) during the first 10 days.

### Obesity-related factors

Obesity-related factors were assessed in both 2018 and 2020. VFA was measured using a bio-impedance-type visceral fat metre (EW-FA90, Panasonic Corporation, Osaka, Japan), which is an authorised medical device in Japan (No. 22500BZX00522000) for non-invasive VFA measurement [31]. The measurements obtained by this device are highly correlated with those obtained using computed tomography (CT) [32], the gold standard for VFA measurement. Morning blood samples were collected from a peripheral vein after ≥ 9 h fasting state. Adiponectin measurement was performed by LSI Medience Co. (Tokyo, Japan) according to their standard operating procedure using latex agglutination turbidimetric immunoassay. Height and body weight were measured, and BMI was calculated from them.

### Other parameters

Data on smoking habits (never/former/current) were obtained through questionnaires prepared for the health check-up. Daily alcohol intake and total energy intake were calculated using the brief-type self-administrated diet history questionnaire (BDHQ) [33, 34]. The BDHQ is a structured questionnaire that contains questions about the intake of approximately 58 foods and beverages, which allows for the estimation of daily alcohol intake and total energy intake.

### Statistical analysis

Given the strong correlation between accelerometer wear time and sedentary time (*r* = 0.80) and the differences in accelerometer wear time, we standardised the sedentary time to 16 h per day of accelerometer wear time using residuals obtained when regressing sedentary time on accelerometer wear time, as previously described [15, 35, 36].

The characteristics of the participants are reported as mean ± standard deviation (SD) or percentage. Owing to their skewed distribution, adiponectin level was log-transformed. Comparisons before and during the COVID-19 pandemic were performed using paired t-tests. The correlation between the two variables was determined using Pearson’s correlation coefficient and partial correlation coefficient. A partial correlation between changes in sedentary time and changes in adiponectin was adjusted by changes in VFA.

Linear regression analysis was used to assess the association of changes in sedentary time or changes in the number of steps with obesity-related factors, which was reported as a regression coefficient (β) and 95% confidence interval (CI) per hour or 1000 steps. In addition to excessive energy intake, VFA is associated with smoking [37] and alcohol intake [38]. Furthermore, it is associated with adiponectin levels [11]. Therefore, we performed regression analysis considering these factors. Model 1 was adjusted for the baseline parameters of sex, age, smoking status, education level, alcohol intake (g/day), total energy intake (kcal/d), sedentary time (h/day) or the number of steps (steps/day), and each obesity-related factor. Model 2 was adjusted for factors in Model 1 plus baseline VFA and change in VFA. Mediation analysis was used to evaluate whether the association between sedentary behaviour and adiponectin was mediated by VFA. Total and direct effects were calculated by multiple-mediated-effects analyses conducted with 5000 bootstrapping repetitions using SPSS PROCESS macro [39]. The variance inflation factors for multicollinearity in the regression analysis were confirmed to be <5. To avoid multicollinearity, changes in sedentary time and changes in the number of steps were not included simultaneously in the model but were separately analysed.

Statistical tests were two-tailed, and statistical significance was set at *P* < 0.05. As interactions by age and sex with changes in sedentary time and number of steps were not statistically significant, pooled analyses were conducted. All analyses were performed using SPSS version 25 software (SPSS Inc., Chicago, IL).

We performed sensitivity analyses using longitudinal data obtained from the health check-up, including in 2019, to confirm that the change in obesity-related factors was due to the COVID-19 pandemic, not due to ageing (see Supplementary Methods). For potential selection bias, we applied an inverse probability weighting approach [40]. Weights were calculated by the inverse of propensity score using logistic regression, considering the difference between the analytical sample and excluded sample (sex, age, alcohol intake, and smoking status). Since simple change scores eliminate autocorrelated error and regression to the mean effects, residualised change scores for sedentary behaviour, number of steps, and obesity-related factors were computed by regressing the values measured during the COVID-19 pandemic onto their respective values measured before the pandemic. The residualised change scores were interpreted as independent of baseline levels [41].

## Results

Table 1 shows the characteristics of the 257 participants before and during the COVID-19 pandemic. Compared to before COVID-19, sedentary time, VFA, and BMI significantly increased, whereas accelerometer wear time and adiponectin significantly decreased during COVID-19 (All *P* < 0.05). Supplementary Fig. 2 shows that these results were independent of covariates. There were no significant changes in alcohol consumption, total energy intake, or the number of steps taken from before to during COVID-19.

**Table 1:** Participant characteristics before and during the COVID-19 pandemic.

	Before (2018)	During (2020)	*P*-value
Age (years)	53.3 (13.6)		
Sex (% women)	63.8		
Smoking status			
Never	65.8		
Former	24.1		
Current	10.1		
Education level			
<10 years	5.06		
10–12 years	56.8		
12 years <	37.3		
others	0.40		
Alcohol intake (g/day)	10.6 (18.4)	10.3 (20.3)	0.742
Energy intake (kcal/day)	1818 (546)	1714 (518)	0.084
Accelerometer wear time (h/day)	15.9 (1.81)	15.4 (2.14)	<0.001
Sedentary time (h/day)^a^	11.1 (1.19)	11.4 (1.18)	<0.001
Average steps (/day)	6580 (2636)	6489 (2850)	0.474
VFA (cm^2^)	80.1 (41.8)	83.4 (43.5)	0.006
BMI (kg/m^2^)	22.6 (3.25)	22.7 (3.21)	0.045
Adiponectin^b,c^ (μg/ml)	2.28 (0.52)	2.21 (0.49)	<0.001

Values are mean (SD) or percentages (*n* = 257).

*VFA* visceral fat area, *BMI* body mass index.

^a^Sedentary time was expressed as the estimated hours of sedentary time per day given as standardised 16 h of accelerometer wear time.

^b^Log transformed values were used.

^c^*n* = 255.

**Fig. 1:**
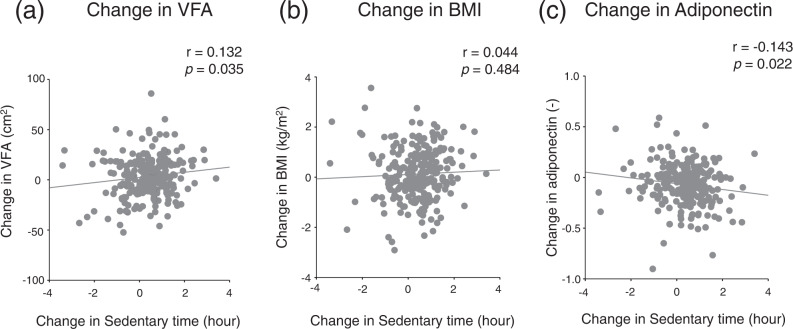
Associations of change in sedentary time with obesity-related factors. Scatter plot with linear regression lines for the relationship between change in sedentary time and (**a**) change in visceral fat area (VFA) and (**b**) change in body mass index (BMI) and (**c**) change in adiponectin levels. Adiponectin level was log-transformed. Correlation between the two variates was determined by Pearson’s correlation coefficient.

Figure 1 shows the relationship between changes in sedentary time and changes in VFA (Fig. 1a), changes in BMI (Fig. 1b), and changes in adiponectin level (Fig. 1c). Changes in sedentary time were found to be significantly associated with changes in VFA (correlation coefficient, *r* = 0.132, *P* = 0.035) and changes in adiponectin (correlation coefficient, *r* = −0.143, *P* = 0.022), whereas changes in sedentary time were not significantly associated with changes in BMI (correlation coefficient, *r* = 0.044, *P* = 0.484). A significant correlation was found between changes in VFA and adiponectin (correlation coefficient, *r* = −0.417, *P* < 0.001). After adjusting for changes in VFA, the association between changes in sedentary time and changes in adiponectin was not significant (partial correlation coefficient, *r* = −0.098, *P* = 0.118).

Table 2 shows the results of the linear regression analysis of the relationship between changes in sedentary time and changes in obesity-related factors. After adjustment for several factors in Model 1, an increase in sedentary time was significantly associated with increased VFA (β = 3.85, 95% CI 1.22 to 6.49, *P* = 0.004) and decreased adiponectin (β = −0.04, 95% CI −0.06 to −0.01, *P* = 0.005), whereas an increase in sedentary time was not significantly associated with increased BMI (β = 0.09, 95% CI −0.05 to 0.23, *P* = 0.213). However, the associations between changes in sedentary time and adiponectin levels did not remain significant after adjusting for changes in VFA (Model 2). Supplementary Table 2 shows the association between changes in the number of steps taken and obesity-related factors. There were no significant associations between changes in the number of steps and changes in VFA and BMI. However, an increase in the number of steps was significantly associated with an increase in adiponectin levels, and this association was independent of changes in VFA. Supplementary Table 3 shows the association between changes in health behaviour and change in obesity-related factors. There were no significant associations between changes in alcohol intake or change in energy intake and changes in VFA and adiponectin, along with the magnitude of these associations, which were lower than sedentary behaviour.

**Table 2:** Association between change in sedentary time and change in obesity-related factors.

	Change in sedentary time					
	Unadjusted		Model 1		Model 2	
Obesity-related factors	β (95% CI)	*P*-value	β (95% CI)	*P*-value	β (95% CI)	*P*-value
Change in VFA	2.57 (0.18, 4.95)	0.035	3.85 (1.22, 6.49)	0.004		
Change in BMI	0.04 (−0.08, 0.17)	0.484	0.09 (−0.05, 0.23)	0.213		
Change in adiponectin^a,b^	−0.03 (−0.05, −0.00)	0.022	−0.04 (−0.06, −0.01)	0.005	−0.02 (−0.05, 0.00)	0.074

Values shown are β (95% confidence intervals). Linear regression models were used. Model 1 was adjusted for baseline parameters of sex, age, smoking status, education level, alcohol intake [g/day], total energy intake [kcal/d], sedentary time [h/day], and each obesity-related factor. Model 2 was adjusted for Model 1 plus baseline VFA and change in VFA.

*VFA* visceral fat area, *BMI* body mass index.

^a^Log-transformed values were used.

^*b*^n = 255.

Supplementary Fig. 3 shows the mediation analysis for the association between change in sedentary behaviour and change in adiponectin by the change in VFA. The total effect of change in sedentary behaviour on change in adiponectin levels was significant (β = −0.04, 95% CI −0.06 to −0.01, *P* = 0.006), however, the direct effect was not significant (β = −0.02, 95% CI −0.05 to 0.002, *P* = 0.074).

**Fig. 2:**
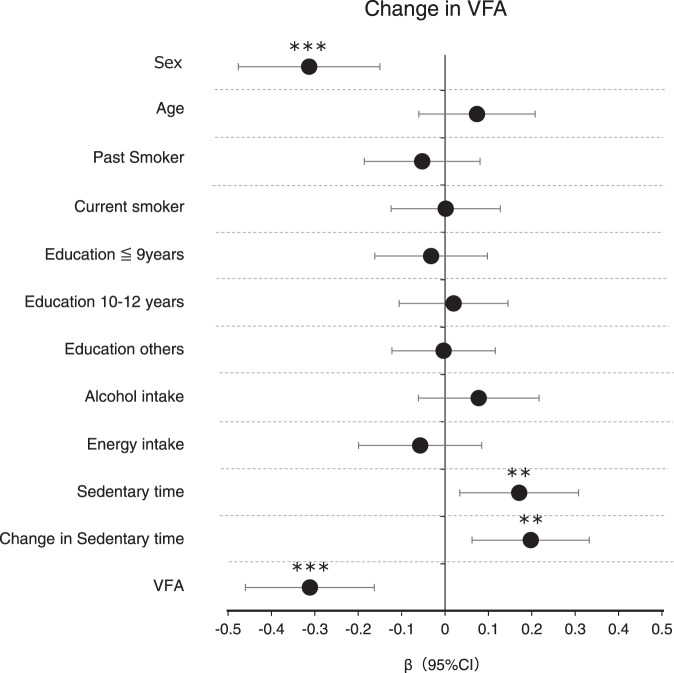
Association of changes in sedentary behaviour and other baseline factors with changes in the visceral fat area (VFA). The values shown are β (95% confidence interval). Explanatory variables were z-scored to allow comparison between variables. VFA visceral fat area. Linear regression models were used with adjustment for baseline parameters of sex, age, smoking status, education level, alcohol intake [g/day], total energy intake [kcal/d], and sedentary time [h/day]. **P < 0.01, ***P < 0.001.

Figure 2 shows a multiple linear regression analysis of the association between changes in sedentary time and VFA, including other explanatory variables. These variables were z-scored to allow comparisons between variables. Higher sedentary time before the COVID-19 pandemic was significantly associated with an increase in VFA; whereas sex (female) and lower VFA were significantly associated with decreased VFA. No significant associations were found for smoking status, education level, alcohol consumption, and total energy intake.

Supplementary Table 4 shows the results of the cross-sectional association between sedentary behaviour and obesity-related factors between two-time points. Compared to that before the COVID-19 pandemic, the magnitude of the association between sedentary behaviour and obesity-related factors was relatively large during the pandemic, especially for VFA.

Results of sensitivity analyses showed that VFA was significantly increased and adiponectin was significantly decreased between 2019 and 2020; however, these were not significantly different between 2018 and 2019 (Supplementary Fig. 4). Supplementary Table 5 shows inverse probability weighted linear regression analyses for the association between changes in sedentary behaviour and obesity-related factors; these results were similar to the original data (Table 2). Supplementary Table 6 shows the association between changes in sedentary behaviour and adiposity using residualised change score; these results were also similar to those in Table 2.

## Discussion

To the best of our knowledge, this is the first study to investigate the association of the COVID-19 pandemic with objectively measured sedentary behaviour, visceral fat and adipocytokines. Sedentary behaviour and VFA were significantly increased, and the increase in sedentary time, as well as baseline long sedentary time, showed a significant association with an increase in VFA. In addition, the association of an increase in sedentary time with decreased adiponectin levels was partly mediated by an increase in VFA. Several studies have reported a significant association between sedentary time and physical activity with obesity during the COVID-19 pandemic [25, 26]. However, these analyses used self-reported data, which may have caused recall bias, and limited simple measurements, such as body weight or BMI. This study contributes to the evidence of the importance of reducing sedentary behaviour to prevent visceral fat accumulation with adipocytokine abnormalities, which causes COVID-19 severity [8, 10].

Some studies have reported that objectively measured sedentary behaviour is associated with visceral fat accumulation in a cross-sectional analysis conducted before the COVID-19 pandemic [15–19]; however, none of the studies has reported a relationship between changes in sedentary time and changes in visceral fat. Under behavioural limitation measures, such as during lockdowns due to the COVID-19 pandemic, the amount of sedentary behaviour could change. Therefore, a longitudinal analysis focusing on this change is required. We analysed two-time points of objectively measured sedentary behaviour before and during the COVID-19 pandemic and found a significant association between an increase in sedentary time and VFA. In addition, a higher baseline sedentary time was associated with an increase in VFA. This result is consistent with longitudinal studies using accelerometer data obtained at one-time point, which have reported that baseline sedentary behaviour is associated with an increase in VFA [42, 43]. Taken together, monitoring and preventing an increase in sedentary behaviour could contribute to the prevention of abdominal obesity under circumstances of behavioural limitation.

Regarding BMI, a systematic review reported its associations with sedentary time [44]. However, we did not observe a significant association, which is consistent with our previous cross-sectional study conducted before the COVID-19 pandemic [15]. One possible explanation of the difference between BMI and VFA is that BMI reflects muscle mass and fat [45] and that an increase in sedentary behaviour was associated with reduced muscle mass [46] so no significant association was seen. Another possible explanation is that visceral fat is more likely to accumulate than subcutaneous fat, which is characteristic of Asian races, including the Japanese [47, 48]. Therefore, in the condition of increased sedentary time, we need to monitor not only BMI but also VFA to reduce the potential risk of COVID-19 severity.

This is the first longitudinal study to analyse the relationship between objectively measured sedentary behaviour and adipocytokine levels. We found a significant association between changes in sedentary time and changes in adiponectin levels; however, these associations did not remain significant after adjusting for VFA. These results are partly consistent with previous cross-sectional studies that reported a significant association between objectively measured sedentary behaviour and adiponectin levels in normal life [19, 20]. However, these studies did not consider the effect of visceral fat. Changes in sedentary time and adiponectin were significantly correlated in bivariate, but not significantly correlated after adjusting for VFA. Mediation analysis revealed that the direct effect of sedentary behaviour on adiponectin was not significant. These results suggest that sedentary behaviour is associated with adipocytokines through the accumulation of visceral fat.

In the present study, there was no significant change in the number of steps taken before and during the COVID-19 pandemic. This report is consistent with a previous study conducted in Japan, where a significant reduction in the number of steps was observed in urban areas but not in rural areas including Aomori Prefecture [23]. Our study participants live in Aomori Prefecture, which may have been less affected by behavioural limitations compared to urban areas in Japan.

Several studies have reported that the COVID-19 pandemic was associated with not only sedentary behaviour but also other health and lifestyle patterns such as dietary habits [12, 13]; however, our data indicated that objectively measured sedentary behaviour increased, while other health behaviours (e.g., alcohol intake and energy intake) were unchanged. Other health behaviours such as a change in energy intake were not significantly associated with a change in the VFA. These results suggested that the association between the changes in sedentary behaviour and obesity-related factors might have become stronger than other health behaviours compared to that before the COVID-19 pandemic. One possible theoretical explanation is that change in sedentary behaviour reduces energy expenditure and muscle mass, contributing to obesity [46]. Further studies are needed to explain these associations.

Analyses between two-time points (before vs. during the COVID-19 pandemic) in this study show that analysing variables assessed as changes (Table 2) provided more statistically significant associations than analysing variables not assessed as changes (Supplementary Table 4). This may be attributed to the nature of adiposity. The cause of adiposity is complicated by a variety of factors. However, the change in adiposity derived from behavioural limitation may be primarily due to decreased energy expenditure caused by increased sedentary behaviour.

The strengths of this study include the analysis of objectively measured data, including sedentary behaviour, VFA, and adipocytokines before and during the COVID-19 pandemic. In addition, activity was measured for ≥7 days (≥10 h/day) with 4 s epochs, which allowed the reflection of actual daily activity [49]. Previous studies have used subjective data from self-reported questionnaires to analyse the association of the COVID-19 pandemic with sedentary time and obesity-related factors; therefore, our study adds important evidence after precisely examining the association. Despite these strengths, our study has several limitations. First, the loss of participants who could not attend the health check-up in 2020, mainly because of the COVID-19 pandemic, could have led to selection bias. The participants included in this study likely reflected a healthier sample. Second, the seasonal difference between the health check-up being conducted in the spring of 2018 and the autumn of 2020 may have influenced some results. Third, there might have been an overestimation of sedentary time since sedentary behaviour was defined based on intensity levels (≤1.5 METs), which cannot distinguish between sitting and standing postures. Finally, as this study was confined to participants from a particular race and region, reproducibility should be confirmed by including participants from different races and/or regions. Behavioural limitation measures, such as lockdown due to the COVID-19 pandemic, in Japan, were relatively mild, and in rural areas, including those in this study, were milder than in urban areas [23], which could have led to an underestimation of the association of the COVID-19 pandemic with sedentary behaviour and physical activity.

## Conclusions

Regardless of the low SARS-CoV-2 infection rate and not having mandatory lockdowns, the COVID-19 pandemic altered sedentary behaviour and obesity-related factors in Japan. In addition, an increase in sedentary behaviour was associated with an increase in VFA, whereas the association of sedentary behaviour with adipocytokines was partly mediated by VFA. Monitoring and managing sedentary behaviour may prevent visceral fat accumulation and thereby improve adipocytokine abnormalities, which contribute to the prevention of COVID-19 severity. Further large-scale longitudinal or interventional studies are required to confirm the effectiveness of reducing sedentary time.

## Supplementary information

Supplementary informations

## Data Availability

The data presented in this study are available on request from the Hirosaki University COI Program Institutional Data Access/Ethics Committee (contact via e-mail: coi@hirosaki-u.ac.jp) for researchers who meet the criteria for access to the data. Researchers must be approved by the research ethics review board at the organisations of their affiliations. The data cannot be shared publicly because of ethical concerns.
