# Supplementary material for: Association of the COVID-19 pandemic with changes in objectively measured sedentary behaviour and adiposity
Source: Int J Obes (Lond). 2023 Feb 16;47(5):375–81. doi: 10.1038/s41366-023-01274-9 (PMC9931562; doi:10.1038/s41366-023-01274-9)
Supplement: Supplementary file 1 — Supplementary informations [file 41366_2023_1274_MOESM1_ESM.docx]

- **Supplementary Methods**
- **Supplementary Figure 1. Flow chart of the study sample.**
- **Supplementary Figure 2. Adjusted participant characteristics before and during the COVID-19 pandemic.**
- **Supplementary Figure 3. Association between change in sedentary behaviour and change in VFA and adiponectin.**
- **Supplementary Figure 4. Impact of the COVID-19 pandemic on obesity-related factors.**
- **Supplementary Table 1. Baseline characteristics of participants included and excluded from the analyses.**
- **Supplementary Table 2. Association between change in the number of steps and change in obesity-related factors.**
- **Supplementary Table 3. Association between change in health behaviour and change in obesity-related factors.**
- **Supplementary Table 4. Cross-sectional association between sedentary behaviour and obesity-related factors between two-time points.**
- **Supplementary Table 5. Association between change in sedentary behaviour and change in obesity-related factors using inverse probability weighting approach.**
- **Supplementary Table 6. Association between change in sedentary behaviour and change in obesity-related factors using residualised change score.**

**Supplementary Methods.**

We performed sensitivity analyses using longitudinal data obtained from the 2018 and 2019 health check-ups before the COVID-19 pandemic and the 2020 health check-up conducted during the COVID-19 pandemic. The study was approved by the Ethics Committee of Hirosaki University School of Medicine (2018-012, 2018-063, 2019–009, and 2020-046-1) and was conducted according to the principles of the Declaration of Helsinki. Written informed consent was obtained from all participants before the study. This study was registered in the University Hospital Medical Information Network ( https://www.umin.ac.jp) prior to the analyses (UMIN ID: UMIN000036741).

In 2019, 1 065 individuals participated in the health check-up from 25 May to 3 June. Among them, 381 individuals who participated in both the 2018 and 2020 health check-ups were enrolled in this study. We excluded 11 individuals had missing data on obesity-related factors. Ultimately, 370 individuals were included in the analysis.

Comparisons of obesity-related factors before and during the COVID-19 pandemic were performed using repeated measures analysis of variance (ANOVA) with post hoc analysis using Bonferroni correction. Statistical tests were two-tailed, and statistical significance was set at *P* < 0.05.

**Supplementary Figure 1. Flow chart of the study sample.**

**
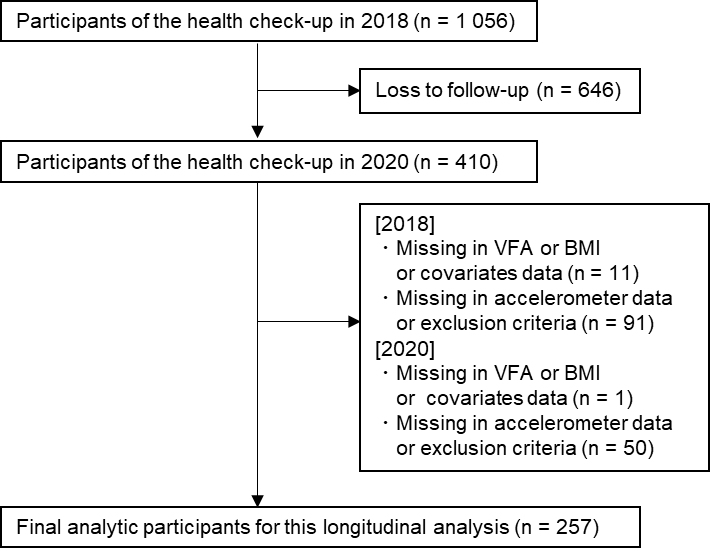
**

A total of 257 participants were included in this study. Abbreviations: VFA, visceral fat area; BMI, body mass index.

**Supplementary Figure 2. Adjusted participant characteristics before and during the COVID-19 pandemic.**

**
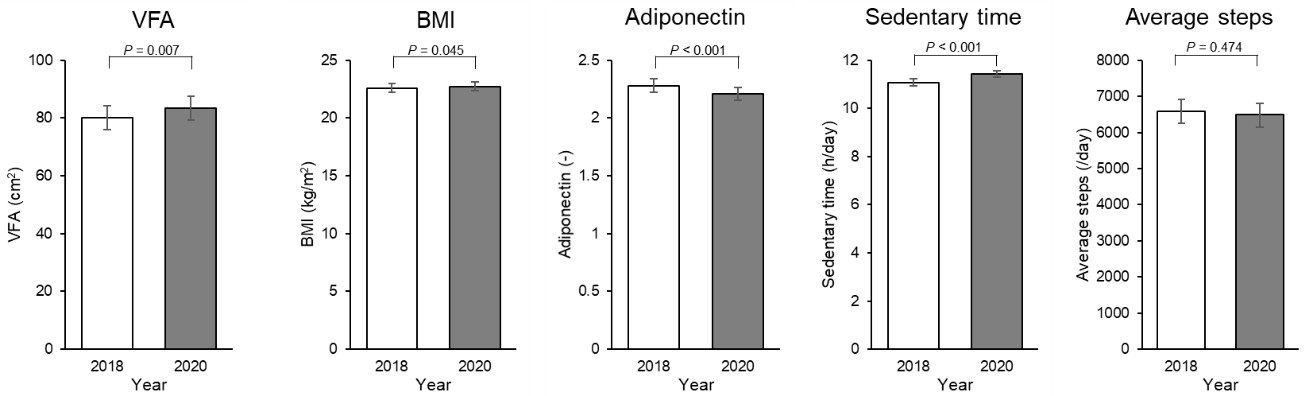
**

Values are presented as adjusted mean (95% confidence interval). Repeated measures analysis of variance with adjusting covariates (sex, age, smoking status, education level, alcohol intake, and total energy intake). Adiponectin level was log-transformed. Abbreviations: VFA, visceral fat area; BMI, body mass index.


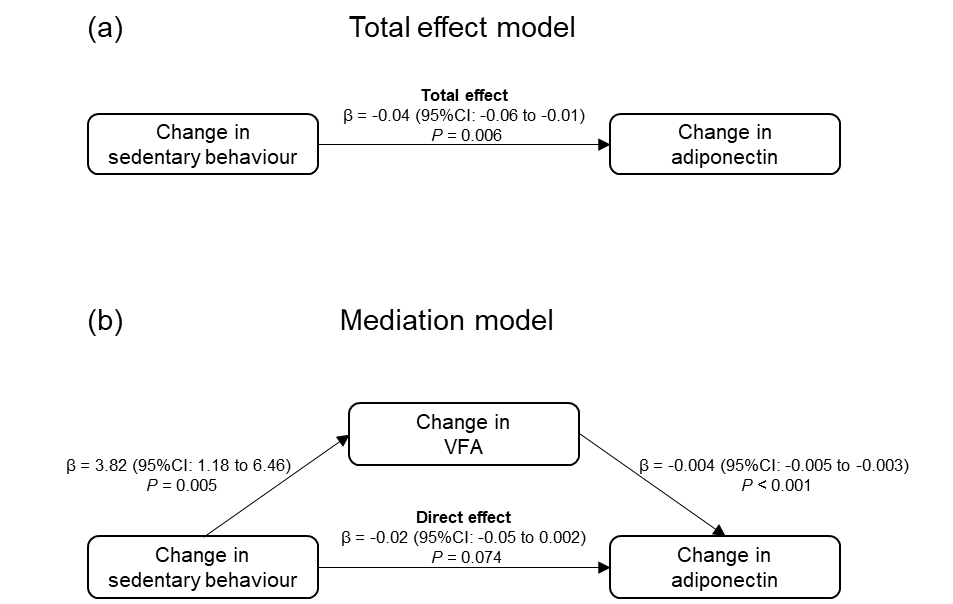
**Supplementary Figure 3. Association between change in sedentary behaviour and change in VFA and adiponectin.**

Values are presented as β (95% confidence interval). Mediation model between change in sedentary behaviour and change in adiponectin by a change in VFA was used, with adjustment for sex, age, smoking status, education level, alcohol intake [g/day], total energy intake [kcal/d], sedentary time [h/day], VFA [cm^2^], and adiponectin level. (a) Total effect model. (b) Mediation effect model. Adiponectin level was log-transformed.

**Supplementary Figure 4. Impact of the COVID-19 pandemic on obesity-related factors.**


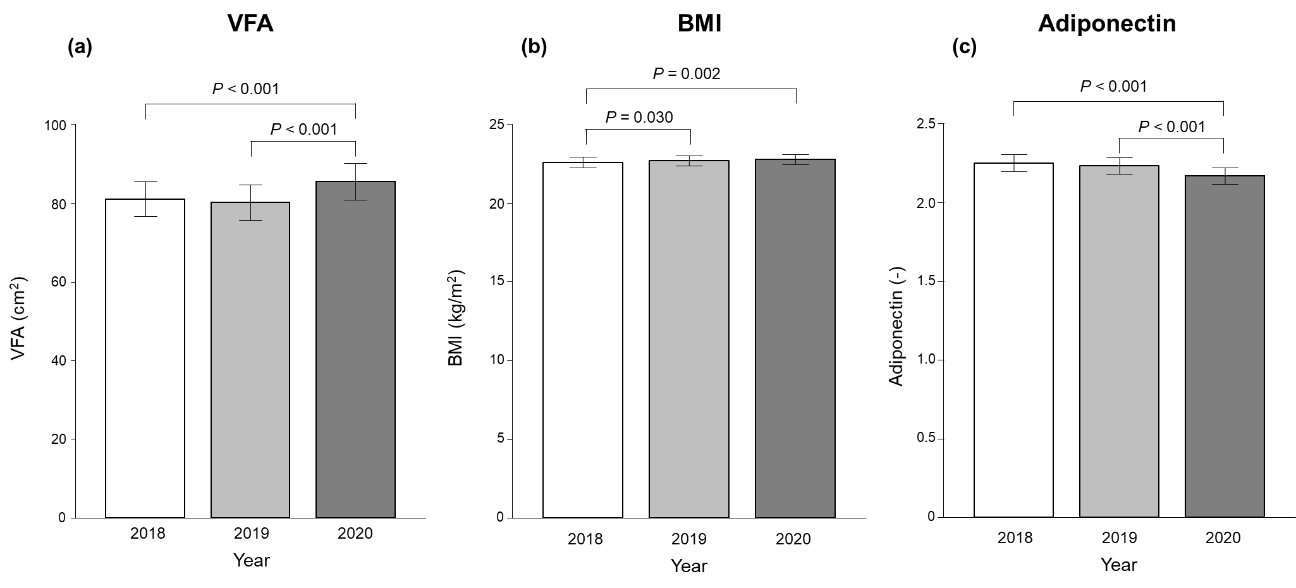


Values are presented as mean (95% confidence interval). Repeated measures analysis of variance (ANOVA) with post hoc analysis using Bonferroni correction. Adiponectin level was log-transformed. Abbreviations: VFA, visceral fat area; BMI, body mass index.

**Supplementary Table 1: Baseline characteristics of participants included and excluded from analyses.**

|  | Included | | Excluded | | *P*-value |
| --- | --- | --- | --- | --- | --- |
|  | n |  | n |  |  |
| Age (years) | 257 | 53.3 (13.6) | 153 | 48.1 (15.5) | <0.001 |
| Sex (% women) | 257 | 63.8 | 153 | 49.7 | 0.005 |
| Alcohol intake (g/day) | 257 | 10.6 (20.3) | 151 | 15.9 (23.6) | 0.001 |
| Change in alcohol intake (g/day) | 257 |  | 151 |  | 0.958 |
| Energy intake (kcal/day) | 257 | 1 818 (546) | 151 | 1 810 (556) | 0.862 |
| Change in energy intake (kcal/day) | 257 |  | 151 |  | 0.064 |
| Smoking status | 257 |  | 153 |  | <0.001 |
| Never |  | 65.8 |  | 54.9 |  |
| Former |  | 24.1 |  | 19.0 |  |
| Current |  | 10.1 |  | 26.1 |  |
| Education level | 257 |  | 153 |  | 0.107 |
| <10 years– |  | 5.06 |  | 11.1 |  |
| 10–12 years |  | 56.8 |  | 51.6 |  |
| 13< years |  | 37.3 |  | 36.6 |  |
| others |  | 0.39 |  | 0.65 |  |
| VFA (cm^2^) | 257 | 80.1 (41.8) | 150 | 82.7 (46.4) | 0.758 |
| Change in VFA (cm^2^) | 257 | 3.30 (19.2) | 149 | 6.27 (17.2) | 0.063 |
| BMI (kg/m^2^) | 257 | 22.6 (3.25) | 145 | 22.8 (3.51) | 0.768 |
| Change in BMI (kg/m^2^) | 257 | 0.13 (1.00) | 145 | 0.27 (1.12) | 0.122 |
| Adiponectin (μg/ml) | 256 | 11.2 (6.03) | 153 | 10.3 (5.01) | 0.307 |
| Change in adiponectin (μg/ml) | 255 | -0.86 (2.22) | 153 | -0.93 (1.96) | 0.436 |

Values are presented as mean (SD) or percentage. Mann-Whitney U tests were used for continuous variables, and Fisher's exact tests were used for categorical variables. Abbreviations: VFA, visceral fat area; BMI, body mass index.

**Supplementary Table 2: Association between change in the number of steps and change in obesity-related factors.**

|  | Change in the number of steps (per 1 000 steps) | | | | | |
| --- | --- | --- | --- | --- | --- | --- |
|  | Unadjusted | | Model 1 | | Model 2 | |
| Change in obesity-related factors | β (95% CI) | *P*-value | β (95% CI) | *P*-value | β (95% CI) | *P*-value |
| Change in VFA | -1.12 (-2.28, 0.04) | 0.058 | -1.07 (-2.28, 0.14) | 0.082 |  |  |
| Change in BMI | -0.003 (-0.06, 0.06) | 0.928 | -0.00 (-0.06, 0.06) | 0.916 |  |  |
| Change in adiponectin^a,b^ | 0.01 (0.00, 0.03) | 0.013 | 0.02 (0.00, 0.03) | 0.010 | 0.01 (0.00, 0.02) | 0.043 |

Values are presented as β (95% confidence interval). Abbreviations: VFA, visceral fat area; BMI, body mass index. Linear regression models were used. Model 1 was adjusted for baseline parameters of sex, age, smoking status, education level, alcohol intake [g/day], total energy intake [kcal/d], number of steps [/day], and each obesity-related factor. Model 2 was adjusted for Model 1 plus baseline VFA and change in VFA. ^a^Log-transformed values were used. ^b^n = 255

**Supplementary Table 3: Association between change in health behaviour and change in obesity-related factors.**

|  | Health behaviour | | | | | |
| --- | --- | --- | --- | --- | --- | --- |
|  | Change in alcohol intake | | Change in energy intake | | Change in sedentary time | |
| Obesity-related factors | standardised β (95% CI) | *P*-value | standardised β (95% CI) | *P*-value | standardised β (95% CI) | *P*-value |
| Change in VFA | 0.07 (-0.07, 0.22) | 0.300 | -0.02 (-0.16, 0.12) | 0.741 | 0.20 (0.06, 0.33) | 0.004 |
| Change in BMI | 0.12 (-0.02, 0.26) | 0.095 | 0.03 (-0.11, 0.18) | 0.654 | 0.09 (-0.05, 0.23) | 0.214 |
| Change in adiponectin^a,b^ | 0.04 (-0.10, 0.18) | 0.584 | 0.12 (-0.02, 0.25) | 0.103 | -0.19 (-0.32, -0.06) | 0.005 |

Values are presented as standardised β (95% confidence interval). Abbreviations: VFA, visceral fat area; BMI, body mass index. Linear regressions models were used with adjustment for baseline parameters of sex, age, smoking status, education level, alcohol intake [g/day], total energy intake [kcal/d], each health behaviour, and each obesity-related factor. ^a^Log-transformed values were used. ^b^n = 255

**Supplementary Table 4: Cross-sectional association between sedentary behaviour and obesity-related factors between two-time points.**

|  | 2018 | |  | 2020 | |
| --- | --- | --- | --- | --- | --- |
|  | Sedentary time | |  | Sedentary time | |
| Obesity-related factors | β (95% CI) | *P*-value |  | β (95% CI) | *P*-value |
| VFA | 2.47 (-1.20, 6.14) | 0.186 |  | 3.61 (-0.14, 7.37) | 0.059 |
| BMI | -0.07 (-0.40, 0.27) | 0.687 |  | 0.07 (-0.26, 0.41) | 0.665 |
| adiponectin^a,b^ | -0.02 (-0.07, 0.03) | 0.424 |  | -0.03 (-0.07, 0.02) | 0.307 |

Values are presented as β (95% confidence interval). Abbreviations: VFA, visceral fat area; BMI, body mass index. Linear regression models with adjustment for sex, age, smoking status, education level, alcohol intake [g/day], and total energy intake [kcal/d]. ^a^Log-transformed values were used. ^b^n = 255

**Supplementary Table 5: Association between change in sedentary behaviour and change in obesity-related factors using** **inverse probability weighting approach.**

|  | Change in sedentary time | | | | | |
| --- | --- | --- | --- | --- | --- | --- |
|  | Unadjusted | | Model 1 | | Model 2 | |
| Change in obesity-related factors | β (95% CI) | *P*-value | β (95% CI) | *P*-value | β (95% CI) | *P*-value |
| Change in VFA | 2.69 (0.26, 5.11) | 0.030 | 3.81 (1.16, 6.45) | 0.005 |  |  |
| Change in BMI | 0.04 (-0.08, 0.17) | 0.484 | 0.08 (-0.06, 0.22) | 0.265 |  |  |
| Change in adiponectin^a,b^ | -0.03 (-0.05, -0.01) | 0.014 | -0.04 (-0.06, -0.01) | 0.009 | -0.02 (-0.05, 0.00) | 0.102 |

Values are presented as β (95% confidence interval). Abbreviations: VFA, visceral fat area; BMI, body mass index. Inverse probability weighted linear regression models were used. Model 1 was adjusted for baseline parameters of sex, age, smoking status, education level, alcohol intake [g/day], total energy intake [kcal/d], sedentary time [h/day], and each obesity-related factor. Model 2 was adjusted for Model 1 plus baseline VFA and change in VFA. ^a^Log-transformed values were used. ^b^n = 255

**Supplementary Table 6: Association between change in sedentary behaviour and change in obesity-related factors using residualised change score.**

|  | Change in sedentary time^a^ | | | | | |
| --- | --- | --- | --- | --- | --- | --- |
|  | Unadjusted | | Model 1 | | Model 2 | |
| Obesity-related factors | β (95% CI) | *P*-value | β (95% CI) | *P*-value | β (95% CI) | *P*-value |
| Change in VFA^a^ | 3.60 (1.00, 6.19) | 0.007 | 3.98 (1.32, 6.64) | 0.003 |  |  |
| Change in BMI^a^ | 0.07 (-0.06, 0.21) | 0.276 | 0.09 (-0.05, 0.23) | 0.211 |  |  |
| Change in adiponectin^a,b,c^ | -0.04 (-0.06, -0.01) | 0.007 | -0.04 (-0.06, -0.01) | 0.005 | -0.02 (-0.05, 0.00) | 0.073 |

Values are presented as β (95% confidence interval). Abbreviations: VFA, visceral fat area; BMI, body mass index. Linear regression models were used. Model 1 was adjusted for baseline parameters of sex, age, smoking status, education level, alcohol intake [g/day], and total energy intake [kcal/d]. Model 2 was adjusted for change in VFAa. ^a^Residualised change score was used. ^b^Log-transformed values were used. ^c^n = 255
